# Sex Differences in Global mRNA Content of Human Skeletal Muscle

**DOI:** 10.1371/journal.pone.0006335

**Published:** 2009-07-22

**Authors:** Amy C. Maher, Minghua H. Fu, Robert J. Isfort, Alex R. Varbanov, Xiaoyan A. Qu, Mark A. Tarnopolsky

**Affiliations:** 1 Department of Medical Sciences, McMaster University, Hamilton, Ontario, Canada; 2 Department of Kinesiology, McMaster University, Hamilton, Ontario, Canada; 3 Department of Pediatrics and Medicine, McMaster University, Hamilton, Ontario, Canada; 4 Procter and Gamble Pharmaceuticals, Mason, Ohio, United States of America; University College Dublin, Ireland

## Abstract

Women oxidize more fat as compared to men during endurance exercise and several groups have shown that the mRNA content of selected genes related to fat oxidation are higher in women (e.g. hormone sensitive lipase, β-hydroxyacyl-CoA dehydrogenase, CD36). One of the possible mechanisms is that women tend to have a higher area percentage of type I skeletal muscle fibers as compared with men. Consequently, we hypothesized that sex would influence the basal mRNA and protein content for genes involved in metabolism and the determination of muscle fiber type. Muscle biopsies from the *vastus lateralis* were collected from healthy men and women. We examined mRNA content globally using Affymetrix GeneChips, and selected genes were examined and/or confirmed by RT-PCR. Furthermore, we examined protein content by Western blot analysis. Stringent gene array analysis revealed 66 differentially expressed genes representing metabolism, mitochondrial function, transport, protein biosynthesis, cell proliferation, signal transduction pathways, transcription and translation. Stringent gene array analysis and RT-PCR confirmed that mRNA for; acyl-coenzyme A acyltransferase 2 (ACAA2), trifunctional protein β (HADHB), catalase, lipoprotein lipase (LPL), and uncoupling protein-2 (UCP-2) were higher in women. Targeted gene analysis revealed that myosin heavy chain I (MHCI), peroxisome proliferator-activated receptor (PPAR)δ were higher in women compared with men. Surprisingly, there were no significant sex based differences in protein content for HADHB, ACAA2, catalase, PPARδ, and MHC1. In conclusion, the differences in the basal mRNA content in resting skeletal muscle suggest that men and women are transcriptionally “primed” for known physiological differences in metabolism however the mechanism behind sex differences in fiber type remains to be determined.

## Introduction

Skeletal muscle is the most abundant tissue in the human body [1], and there are major differences between women and men in energy metabolism, fiber type composition, and contractile speed [2], [3], [4]; however, the mechanisms behind these differences are unknown. A number of groups have reported differences in exercise related substrate metabolism between men and women, specifically that women oxidize more lipid and less carbohydrate than men [5], [6], [7], [8], [9], [10], [11]. Similar studies in rats have also found gender differences in lipid metabolism, specifically that female rats have greater lipid oxidation and muscle lipoprotein lipase (LPL) activity, as compared with male rats [12], [13]. Furthermore, when female rats are oophorectomized, lipid oxidation is lower during endurance exercise [14]. The exact mechanisms behind such observations are unclear; however, sex differences in mRNA content and protein expression appear to be directionally consistent with the observed metabolic differences present during exercise. Specifically, women compared to age and fitness matched men have higher mRNA content for LPL [15], membrane fatty acid transport protein 1 (FATm) [16], FAT/CD36 transporter and plasma membrane fatty acid binding protein (FABPpm) [15], citrate synthase [17], β-hydroxyacyl-CoA dehydrogenase (β-HAD) [17], and hormone sensitive lipase [11]. On the whole body level, women show greater lipolysis [5], [18], and greater uptake of plasma free fatty acids [18], and use more intramuscular triacylglycerol [11], [19], [20] than men.

Futhermore, a sex difference in muscle fiber composition has been found in different skeletal muscles, such as, *erector spinae* [21], *internal and external intercostal, latissimus dorsi* [22], *biceps brachii* [23], *vastus medialis* [24], *and vastus lateralis* [5], [25], [26], [27], [28], [29], [30]. Although studies concerning sex differences in skeletal muscle fiber type have shown inconsistent results [4], [25], [26], [28], [29], [31], [32], women generally have a greater relative type I fiber area [4], [5], [26], [27], [30], a smaller relative area of type II fibers and a greater percentage area of type I fibers [4], [5], [26], [27], [28], [32]. The potential molecular mechanisms behind these sex differences have not been evaluated for the mRNA species of the genes involved in muscle development, or fiber-type determination and differentiation.

The molecular mechanisms for the observed differences in substrate oxidation and fiber type between men and women are unknown. In this study we used a targeted approach (RT-PCR) to evaluate mRNA species of the genes involved in muscle development, fiber-type determination and differentiation, and a microarray comparison to evaluate the many more potential mRNA species that are required for lipid metabolism and fiber type in human skeletal muscle, which may yield important data for understanding potential novel mechanisms. Gene array technology has provided a rapid and efficient way to screen a large number of mRNAs in order to identify potential targets and pathways for further research. Recently, several groups have used gene arrays to identify novel transcriptional programs related to human muscle repair, inflammation, protein synthesis and cellular control in skeletal muscle after various interventions such as exercise [33], [34], [35], immobilization [36], and drug treatments [37]. A few groups have done targeted gene expression profiles to examine sex differences in humans [38], [39] and mice [40]. In this study, skeletal muscle biopsies from healthy, young men and women were analyzed for mRNA abundance of over 23,000 genes by Affymetrix gene array analysis with an *a priori* hypothesis that mRNA species involved in lipid oxidation, muscle development, and fiber-type determination and differentiation would be different in women compared with men. Furthermore, we hypothesized that this global analysis would identify novel mRNA targets that are relevant to biological pathways that differ in skeletal muscle between men and women.

## Materials and Methods

### Participants

These studies were approved by the Human Research Ethics Board of McMaster University. The present study used muscle samples from two different studies that recruited subjects using identical criteria and subject characteristics were not significantly different (Table 1). All subjects were between the ages of 18 and 35, healthy, recreationally active, and non-smokers. Highly trained athletes were excluded from the studies. All female participants were eumenorrheic. The present study only compared muscle samples collected at baseline (before manipulation) thus termed “resting muscle” from the two studies. In *study 1*, 14 women and 13 men volunteered to participate but we only used samples from 12 women and 12 men. Subject characteristics have been previously published [30] but can be viewed in Table 1. In *study 2*, 24 healthy, recreationally active men (n = 11) and women (n = 13) participated [41]. Data from these subjects regarding diet, CHO, protein and fat oxidation has been previously reported [41], and demonstrated that women had higher fat oxidation and lower protein and carbohydrate oxidation during endurance exercise as compared with men. The muscle samples collected pre-exercise were used for the present study and only samples from women in the follicular phase of their menstrual cycle were used as there was little difference in mRNA content of metabolic related genes between follicular and luteal phase women (Figure S1) compared to differences due to sex (Figure S2) (unpublished data). *Study 1* samples were used to acquire the gene array data, histochemical data is a combination of a representative subset of subjects from both studies, and RNA and protein data was acquired from *study 2* samples. A comparison of subject characteristics is reported in Table 1. All study participants gave written consent to participate in the study.

**Table 1:** Subject characteristics from study #1 and study #2.

	Study #1		Study #2	
	Men (n = 12)	Women (n = 12)	Men (n = 11)	Women (n = 13)
Age (yr)	21±1	22±1	21±1	22±2
Weight (kg)	79±4	61±2*	80±3	63±2*
Height (cm)	179±2	164±1*	178±1	165±1*
BF (%)	18±1	25±1*	19±5	29±5*
FFM (kg)	64±3	44±1*	59±1	52±1*
BMI	25±1	23±1	25±1	23±1
VO2peak (ml*kg body wt-1*min-1)	NA	NA	45±1	39±2*
(ml*kg FFM-1*min-1)	NA	NA	56±1	54±3
Menstral cycle		7 = Fol, 5 = Lut		Fol
Oral Contraceptive use		6 = OC, 6 = NOC		6 = OC, 7 = NOC
Feeding state	Boost® 2 hrs before biopsy		Fasted (10–12 hrs)	

* significant difference between men and women for each study (P<0.05). There is no significant difference between the men or the women in study 1 compared with study 2. BF; body fat, BMI; body mass index, Fol; follicular phase, Lut; luteal phase, OC; oral contraceptives.

### Acquisition of muscle samples

Muscle samples were obtained from the *vastus lateralis* muscle, ∼15 cm proximal to the lateral knee joint line, using a custom suction-modified Bergstrom needle. Biopsies were taken at rest and all subjects refrained from any exercise for at least 3 days before the muscle biopsy. All biopsies were completed in the morning. Approximately 60 mg of muscle tissue was divided and snap-frozen in liquid nitrogen and stored in a −80°C freezer for RNA and protein analysis.

### Histochemical analysis

Histochemical analyses were conducted on samples from *study 1and 2*, as described by Yasuda *et al*. [30]. Briefly, the OCT mounted muscle samples were serially sectioned to 10 µm thickness, and slides were preincubated at a pH value of 4.60 in 50 mM potassium acetate, 17.5 mM calcium chloride for 7 min. Slides were rinsed with distilled, deionized water (ddH20) between each of the following steps. Slides were incubated in 3 mM ATP using an alkaline solution (75 mM glycine, 40.5 mM calcium chloride, 75 mM NaCl, 67.5 mM NaOH, pH 9.4) for 45 min at 37°C with agitation at regular intervals. They were incubated consecutively in 1% CaCl_2_ and 2% CoCl_2_ for 3 min, and then incubated in 1% ammonium sulphide for 30 s at room temperature. Sections were photographed at 200× magnifications with a microscope (Olympus America Inc., Melville, NY) in conjunction with a SPOT digital camera (Model: SP401-115, SPOT Diagnostic Instruments Inc., MI). Based on the staining intensity at pH 4.60, the three fiber types were classified as Type I (dark), IIa (light) and IIx (intermediate). Cross-sectional areas of the muscle fibers (µm^2^) were determined using an image analysis program (Image Pro, V6.0, Media Cybernetics Inc., Silver Spring, MD).

### Microarray Preparation

Muscle samples from *study 1* were used to extract RNA, and the RNA was prepared according to Affymetrix recommendations (Santa Clara, CA). In brief, muscle samples were homogenized in TRIzol Reagent (Life Technologies, Rockville, MD) by shaking in a mixer mill with tungsten carbide beads as recommended by the manufacturer (Qiagen, Life Technologies, Rockville, MD). RNA was purified using an RNeasy Mini Kit (Qiagen, Chatsworth, CA). Ten micrograms of purified RNA was reverse transcribed using SuperScript II Reverse Transcriptase and T7-(dT)_24_ primer followed by second strand DNA synthesis as per the manufacturer's instructions (Life Technologies, Rockville, MD). Contaminants were removed from the samples by phenol-chloroform-isoamyl alcohol extraction, and the clean cDNA was recovered by ethanol precipitation, using a RNA Transcript Labeling Kit (Enzo Diagnostics, Farmingdale, NY), it was further converted into biotin-labeled cRNA, as per manufacturer's instructions. Moreover, cRNA was purified using a RNeasy Mini Kit (Quiagen, Life Technologies, Rockville, MD) and fragmented in pieces <200 bases by incubation in fragmentation buffer. Samples were stored at −20°C until hybridization.

Samples were hybridized on human HG-U133 Plus 2.0 Arrays using protocols as recommended by Affymetrix (Affymetrix, Santa Clara, CA). In brief, 24 gene chips were used to compare 12 women to 12 men. Biotinylated cRNA was hybridized for 16 h at 45°C in a GeneChip Hybridization Oven 640. Arrays were placed in a GeneChip Fluidic Station 400 for a series of washes, followed by incubation with streptavidin-conjugated phycoerythrin. Finally, arrays were scanned with a GeneArray Scanner (Agilent, Palo Alta, CA) and analyzed using GeneChip Analysis software (Affymetrix, Santa Clara, CA).

### Statistical Analysis of MicroArray

The statistical analysis was based on the Affymetrix signal (MAS 5.0 algorithm). Exploratory statistical tools were used to check data quality. There were no quality control problems with the data. The data was then filtered based on the given algorithms for gene content levels, which filtered out genes that have low content levels compared to background (Affymetrix Absent Calls). When a minimum of 19 out of 24 gene replicates read as Affymetrix Absent Calls, the gene is filtered out. An ANOVA model with log (base 2) of the Affymetrix signal as a response is fitted for each one of the genes that are not filtered based on Affymetrix Absent Calls. Significant differential content is calculated by the NLOGP (−log_10_(P-value)). A gene was considered to be differentially expressed if the NLOGP measure was greater than the 4, and the fold change was at least 1.2. Table S1. Proctor and Gamble use these criteria for their Affymetrix gene array analyses [42]. The negative log of the *p* value (NLOGP) was calculated from (−log_10_ (NLOGP 4 = *p* value threshold of 0.0001), where the *p* value is a summary measure of the statistical significance for the corresponding comparison.

### Gene annotation and functional analysis

Gene function and characterization information was obtained from the Affymetrix website: http://www.affymetrix.com/analysis/index.affx. Further description was obtained from various databases at the National Center for Biotechnology Information. iPath™ (Invitrogen) was used to compared differentially regulated genes against 225 signaling and metabolic human biological pathway maps created for Invitrogen™ by GeneGo. (http://escience.invitrogen.com/ipath). Differentially regulated gene were also run against a Connectivity Map, which is a gene expression database with statistical scoring algorithm to link drug treatment, genes, and diseases.

All gene array data can be accessed via Gene Expression Omnibus (GEO) http://www.ncbi.nlm.nih.gov/geo/. Accession #GSE14901. The data used for this study were the PRECAST values (Table S1) only from a larger study “Limb immobilization induces a coordinate down-regulation of mitochondrial and other metabolic pathways in men and women”.

### RNA isolation for RT-PCR

Muscle biopsy samples from *study 2* were used to isolated RNA as described previously[43]. Briefly, frozen muscle was thawed in a Tenbroeck homogenizer with 1 ml of TRIzol reagent (Invitrogen, Carlsbad, CA) and homogenized on ice. The homogenate was extracted with 200 µl of chloroform. The aqueous phase was removed and the RNA was precipitated at room temperature using 500 µl of iso-propanol and washed twice with 75% ethanol. The final RNA pellet was air dried at room temperature and resuspended in 14 µl ddH_2_O and treated with DNaseI. The RNA samples were quantified by spectrophotometer (A_260_/A_280_ ≥1.5) and the quality was assessed by agarose gel electrophoresis.

### TaqMan® Real-time RT-PCR

TaqMan® real-time RT-PCR was conducted on total RNA. Duplex RT-PCR was performed using an iCycler real-time PCR system (Bio-Rad Laboratories, Hercules, CA). One-step TaqMan® RT-PCR Master Mix Reagent (Roch, Branchburg, New Jersey) was combined with RNA, target gene primers and probes, and internal standard gene primers and probes in the same reaction. Specific primers and probes to each target gene were designed based on the cDNA sequence in GenBank (http://www.ncbi.nlm.nih.gov/entrez/query.fcgi) with primer 3 designer (http://frodo.wi.mit.edu/cgi-bin/primer3/primer3www.cgi.). Their specificity was monitored using Blast (http://www.ncbi.nlm.nih.gov/BLAST/). Primer sequences are listed in Table 2.

**Table 2:** Primer Sequences.

Gene Name	Forward Primer	Reverse Primer
beta2-microglobulin	ggctatccagcgtactccaa	gatgaaacccagacacatagca
Catalase	actgaggtccaccctgactac	tcgcattcttaggcttctca
Lipoprotein Lipase (LPL)	gaaaggcacctgcggtatt	catgccgttctttgttctgta
Uncoupling protein-2 (UCP-2)	tcatcacctttcctctggatac	agaatggtgcccatcacac
Acyl-coenzyme A acyltransferase-2 (ACAT2)	ggcaggagaagcaagatga	gcaccattgaaggaacctatg
Aldehyde dehydrogenase 1 family member A1 (ALDH1A1)	cgccagacttacctgtcctact	ctcctcagttgcaggattaaag
Trifunctional protein β subunit (HADHB)	aaacaagcaatgtggctagaga	ggcttggttggcagagatac
Myosin Heavy Chain I (MHCI)	cctggaacatctggagacct	agctgctttcggaccttct
Myosin Heavy Chain IIa (MHCIIa)	caatctagctaaattccgcaagc	tcacttatgacttttgtgtgtgaacct
Myosin Heavy Chain IIx (MHCIIx)	aaatggtggaaagaagagagtcc	aatacagcttcatccagggc
PGC-1alpha	ttgctaaacgactccgagaa	tgcaaagttccctctctgct
PPARdelta	actgagttcgccaagagcat	gtgcacgccatacttgagaa
Myostatin	gacccgtcgagactcctaca	aataccagtgcctgggttca

* All primer sequences are shown 5′ to 3′, left to right.

All probes were dual-labeled with fluorophores, with a fluorescent reporter dye at the 5′ end (FAM, TET, HEX or TAMRA) and a corresponding quencher dye at its 3′ end (3BHQ-1 or 3BHQ-2). Human β2- microglobulin (β2-M) was used as an internal standard as there was no significant sex difference in expression (data not shown). All samples were run in duplicate simultaneously with RNA- and RT-negative controls. Fluorescence emission was detected through a filter corresponding to the reporter dye at the 5′end of each probe, and C_T_ was automatically calculated and displayed.

### Western blot analysis

Thirty mg of tissue was used for protein content analysis. Muscle tissue was homogenized in a phosphate lysis buffer; 50 mM K_2_HPO_4_, 1 mM EDTA, pH 7.4, 0.1 mM DDT, PhosSTOP (Roach Diagnostics, Mannheim, Germany), Protease inhibitor cocktail tablets (Roach). Protein concentrations were calculated by Bradford assay (Biorad) and equal amounts of protein were boiled in Laemmli buffer, resolved by SDS-PAGE, transferred to nitrocellulose paper and immunoblotted with desired antibodies. Primary antibodies; HADHB, ACAA2, catalase, PPARδ, MHC I, MHC II and β-actin were all purchased from (Abcam, Cambridge, MA). Secondary antibodies conjugated to horseradish peroxidase (Amersham Bioscience, UK) and specific antibody binding was detected using the chemiluminescence detection reagent ECL+ (Amersham BioScience, UK). Scanned films were analyzed using ImageJ 1.40 software (Wayne Rasband National Institute of Health, USA).

### Statistical analysis

All statistical analyses, for mRNA content of the genes, was performed on linear data 2^−CT^ for evaluation of internal standards, 2^−ΔCT^ for target gene normalized with internal reference [44]. Data on sex differences about target gene mRNA content were analyzed using a Student's *t* test. All results from evaluation of target gene are expressed as mean±SEM, using 2^−ΔCT^. Western blot data was normalized by the loading control (β-actin) and a Student's *t* test was preformed to test for a difference between men and women. Data are presented as means±SEM. The data regarding muscle fiber composition were analyzed using a single factor ANOVA and expressed as mean±SEM. All analyses were done using statistics software (Statistica version 5.0; Statsoft, Tulsa, OK). Statistical significance was set at α≤0.05.

## Results

### Sex alters mRNA content in skeletal muscle; microarray

The mRNA abundance in skeletal muscle between men and women was significantly different for 66 genes (using a stringent NLOGP≥4, fold-change>1.2), after Y-linked genes were removed (Table 3; GEO accession #GSE14901). Of these 66 genes, 49 genes have known functions in metabolism, mitochondrial function, transport, protein biosynthesis, cell proliferation, signal transduction pathways, transcription and translation (Table 3). Conversely, 17 of the genes identified in the current analysis do not have known functions. Of the 49 genes with known function, women had higher content of 25 genes (mean 1.7±0.4), compared to men. Subsequently, women have lower content of 24 genes (mean −2.1±2.0), compared to men.

**Table 3:** Differential expression of mRNA in women vs men.

Gene Name	Symbol	Fold Change	NLOGP	Biological Process
**Metabolism**				
hydroxyacyl-Coenzyme A dehydrogenase/3-ketoacyl-Coenzyme A thiolase/enoyl-Coenzyme A hydratase (trifunctional protein), beta subunit	HADHB	1.32	4.00	lipid metabolism, fatty acid metabolism, fatty acid beta-oxidation
acetyl-Coenzyme A acyltransferase 2 (mitochondrial 3-oxoacyl-Coenzyme A thiolase)	ACAA2	1.61	5.20	lipid metabolism, fatty acid metabolism, cholesterol biosynthesis
lipoprotein lipase	LPL	1.85	4.30	fatty acid metabolism, circulation, lipid catabolism
aldehyde dehydrogenase 2 family (mitochondrial)	ALDH2	1.46	4.70	carbohydrate metabolism, alcohol metabolism
argininosuccinate synthetase 1	ASS1	2.19	6.60	urea cycle, arginine biosynthesis, amino acid biosynthesis
aldehyde dehydrogenase 1 family, member A1	ALDH1A1	−1.88	6.00	aldehyde metabolism
**Mitochondrial function/oxidative stress**				
catalase	CAT	1.7	6.60	electron transport, response to oxidative stress, hydrogen peroxide catabolism
uncoupling protein 2 (mitochondrial, proton carrier)	UCP2	1.5	4.20	proton transport in the mitochondria
ATP synthase mitochondrial F1 complex assembly factor 1	ATPAF1	−1.49	4.00	protein complex assembly
**Transport**				
solute carrier family 25, member 34	SLC25A34	2.37	4.20	transport (mitochondrial carrier)
solute carrier family 1 (glutamate/neutral amino acid transporter), member 4	SLC1A4	−2.2	4.80	dicarboxylic acid transport, neutral amino acid transport
**Signal transduction**				
Rap guanine nucleotide exchange factor (GEF) 2	RAPGEF2	1.21	4.00	intracellular signal transduction, MAPK cascade, cAMP-mediated signaling ,
transducin-like enhancer of split 1 (E(sp1) homolog, Drosophila)	TLE1	1.7	4.00	regulation of transcription, signal transduction, frizzled signaling pathway
kalirin, RhoGEF kinase	KALRN	1.31	4.00	protein amino acid phosphorylation, signal transduction, vesicle-mediated transport
amyloid beta (A4) precursor-like protein 2	APLP2	1.32	5.60	G-protein coupled receptor protein signaling pathway
growth factor receptor-bound protein 10	GRB10	2.41	7.80	intracellular signaling cascade , cell-cell signaling , insulin receptor signaling pathway
Cyclin-dependent kinase inhibitor 1C (p57, Kip2)	CDKN1C	1.71	5.20	regulation of cyclin-dependent protein kinase activity , G1 phase of mitotic cell cycle
family with sequence similarity 13, member A1	FAM13A1	1.32	4.20	signal transduction
mitogen-activated protein kinase 6	MAPK6	−1.34	4.00	protein amino acid phosphorylation , cell cycle , signal transduction
WW domain containing E3 ubiquitin protein ligase 1	WWP1	−1.52	7.40	signal transduction, negative regulation of transcription , protein ubiquitination, protein modification , ubiquitin cycle
Rho guanine nucleotide exchange factor (GEF) 10-like	ARHGEF10L	−1.31	4.10	regulation of Rho protein signal transduction
**Transcription**				
Sine oculis homeobox homolog 1 (Drosophila)	SIX1	1.34	4.00	regulation of transcription, muscle development
small nuclear ribonucleoprotein polypeptide N, SNRPN upstream reading frame	SNRPN, SNURF	−1.6	5.90	mRNA metabolism
nuclear receptor interacting protein 1	NRIP1	−1.59	4.60	negative and positive regulation of transcription from RNA polymerase II promoter , androgen receptor signaling pathway
TBC1 (tre-2/USP6, BUB2, cdc16) domain family, member 1	TBC1D1	−1.45	4.40	DNA metabolism , chromosome organization and biogenesis
cyclin H	CCNH	−1.21	4.80	regulation of cyclin-dependent protein kinase activity , DNA repair , regulation of transcription
LAG1 longevity assurance homolog 6 (S. cerevisiae)	LASS6	−1.82	4.10	regulation of transcription, lipid biosynthesis
iroquois homeobox protein 3	IRX3	−10.92	11.40	regulation of transcription
zinc finger protein 33A	ZNF33A	−1.57	4.00	regulation of transcription
**Protein Biosynthesis and Translation**				
eukaryotic translation initiation factor 1A, X-linked	EIF1AX	1.38	7.30	protein biosynthesis, translational initiation
eukaryotic translation initiation factor 2, subunit 3 gamma, 52 kDa	EIF2S3	1.45	7.20	protein biosynthesis
FK506 binding protein 9, 63 kDa	FKBP9	1.32	4.00	protein folding
similar to Caspase-4 precursor (CASP-4) (ICH-2 protease) (TX protease) (ICE(rel)-II)	LOC648470	1.9	4.50	proteolysis
JTV1 gene	JTV1	−1.42	5.80	protein biosynthesis
ubiquitin specific peptidase 31	USP31	−1.42	4.10	ubiquitin-dependent protein catabolism
ring finger and CHY zinc finger domain containing 1	RCHY1	−1.31	5.10	ubiquitin cycle
ADP-ribosylhydrolase like 1	ADPRHL1	−1.61	4.70	protein amino acid ADP-ribosylation
**Cell Proliferation**				
zinc finger protein 36, C3H type-like 2	ZFP36L2	1.48	4.80	cell proliferation
monocyte to macrophage differentiation-associated	MMD	1.67	4.10	cytolysis
angiopoietin 1	ANGPT1	1.74	4.30	angiogenesis, signal transduction, cell differentiation, development
plexin C1	PLXNC1	1.94	4.70	cell adhesion, development
cell division cycle associated 7-like	CDCA7L, RAM2	2.45	4.40	positive regulation of cell proliferation
tumor protein D52	TPD52	−1.72	5.70	morphogenesis, B cell differentiation, secretion
Down syndrome critical region gene 1-like 1	DSCR1L1	−1.67	5.90	central nervous system development, calcium-mediated signaling
cytokine induced apoptosis inhibitor 1	CIAPIN1	−1.31	4.90	apoptosis, anti-apoptosis
CD24 molecule	CD24	−4.19	7.00	humoral immune response
dishevelled associated activator of morphogenesis 2	DAAM2	−1.71	4.20	actin cytoskeleton organization and biogenesis
attractin-like 1	ATRNL1	−1.83	4.80	development
spectrin, beta, non-erythrocytic 1	SPTBN1	−1.52	5.30	barbed-end actin filament capping
**UNKNOWN**				
ubiquitously transcribed tetratricopeptide repeat, X chromosome	UTX	2.13	9.35	---
zinc finger, BED-type containing 5	ZBED5	−1.31	4.30	---
Nedd4 family interacting protein 2	NDFIP2	−1.4	4.60	---
chromosome 2 open reading frame 25	C2orf25	−1.23	4.00	---
tryptophan rich basic protein	WRB	−1.47	6.00	---
PQ loop repeat containing 3	PQLC3	1.77	4.30	---
CDNA FLJ25488 fis, clone CBR00232	---	−1.34	5.20	---
family with sequence similarity 79, member B	FAM79B	5.94	8.80	---
X (inactive)-specific transcript	XIST	192.07	32.77	---
CDNA FLJ33569 fis, clone BRAMY2010317	---	−1.47	4.00	---
hypothetical protein	LOC387882	3.04	5.30	---
chromosome 8 open reading frame 22	C8orf22	2	4.20	---
chromosome Y open reading frame 15A	CYorf15A	−2.01	5.10	---
KIAA1155 protein	KIAA1155	1.71	5.10	---
Prader-Willi syndrome chromosome region 1	PWCR1	−1.61	5.00	---
Heparan-alpha-glucosaminide N-acetyltransferase, similar to transmembrane protein 76	HGSNAT, LOC643642	1.53	5.30	---
hypothetical gene CG018	CG018	1.61	6.60	---

* Microarray significance >1.2 fold increase or decrease. NLOGP, negative log of the *p* value, >4.0. N = 12 men and 12 women.

iPath™ (Invitrogen) was used to compared differentially regulated genes against 225 signaling and metabolic human biological pathway maps, and these gene were found to be involved in 39 different signal transduction pathways. Differentially regulated genes were also run against a Connectivity Map to compare the expression profile to that of known drug treatments, genes, and diseases. Interestingly, differentially expressed genes between genders were found to share a similar expression pattern with the transcript profiling of estradiol (which is estrogenic), genistein (which could be estrogenic or anti-estrogenic) and tretinoin (a vitamin A derivative) drug treatments.

### Sex alters mRNA content of genes involved metabolism and mitochondrial biogenesis

Sex altered content of six genes involved in metabolism and three genes involved in the function of mitochondria and energy production (Table 3). HADHB (trifunctional protein β), ACAA2, LPL, UCP-2, catalase, aldehyde dehydrogenase 2 family (ALDH2), and argininosuccinate synthase 1 (ASS1) all had higher mRNA content in women compared to men by 1.3- to 2.2-fold. ALDH1A1 and ATP synthase mitochondrial F1 complex assembly factor 1 were significantly lower in women compared to men by 1.9 fold and 1.5-fold respectively (Table 3).

**Figure 1:**
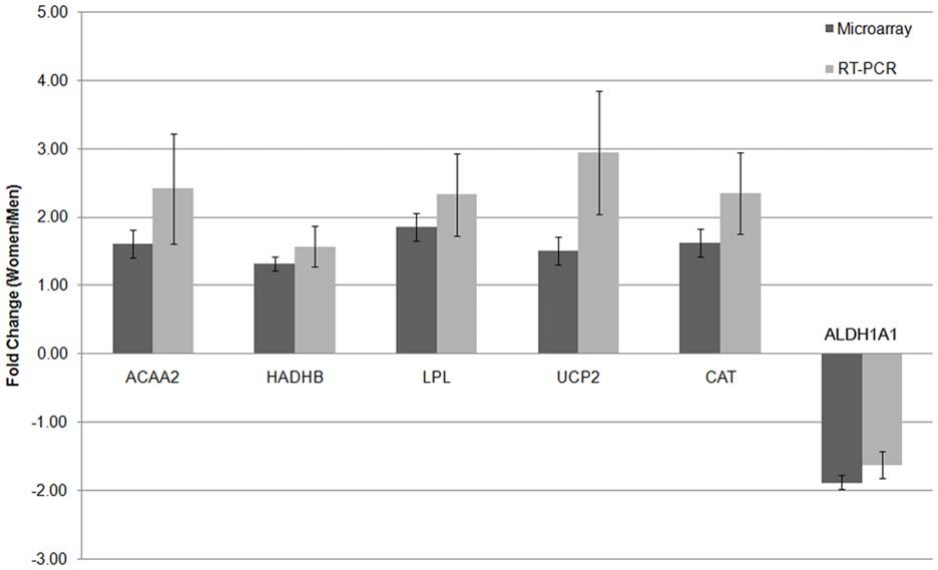
Comparison of microarray data to RT-PCR results for 6 genes found to be significantly different between men and women. Shown as fold change in women versus men. N (microarray) = 12 men, 12 women; N (RT-PCR) = 12 men, 12 women.

**Figure 2:**
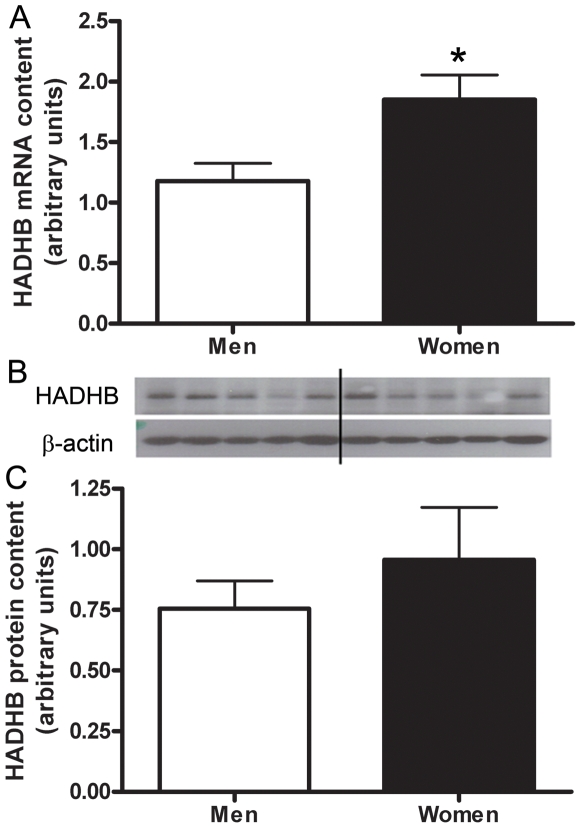
Sex differences in HADHB. Differences in HADHB muscle mRNA content between men and women shown by Real time RT-PCR, adjusted to β2-M mRNA (A). Protein content of HADHB in skeletal muscle of men and women, adjusted to β-actin (B,C). Lanes 1–4 are men, lanes 5–8 women, representative of all blots. N = 12 men and 12 women. *P<0.05.

**Figure 3:**
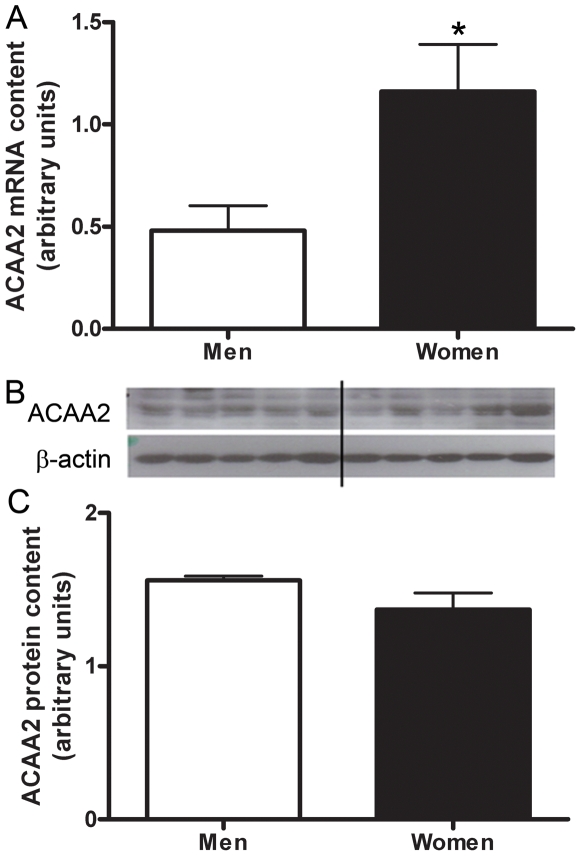
Sex differences in ACAA2. Differences in ACAA2 muscle mRNA content between men and women shown by Real time RT-PCR, adjusted to β2-M mRNA (A). Protein content of ACAA2 in skeletal muscle of men and women, adjusted to β-actin (B,C). Lanes 1–4 are men, lanes 5–8 women, representative of all blots. N = 12 men and 12 women. *P<0.05.

The mRNA content of genes involved in lipid metabolism, HADHB, ACAA2, and catalase, were confirmed by real time RT-PCR (Figure 1, 2a, 3a, 4a) using *study 2* samples. The mRNA content of UCP-2, LPL, and ALDH1A1 were also confirmed by real time RT-PCR (Figure 1, 5), and fold-change of the RT-PCR results correlated to the fold-change reported by the microarray (Figure 1).

**Figure 4:**
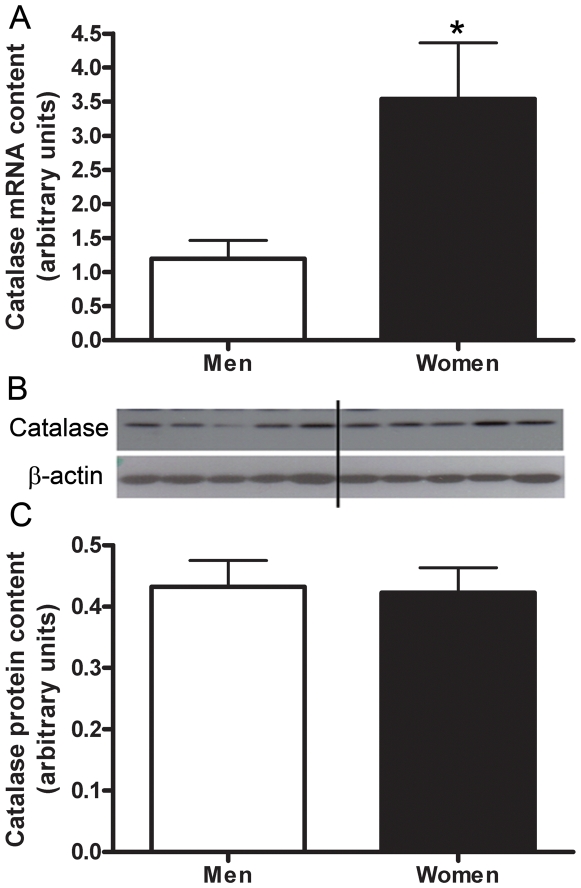
Sex differences in catalase. Differences in Catalase mRNA content between men and women shown by Real time RT-PCR, adjusted to β2-M mRNA (A). Protein content of catalase in skeletal muscle of men and women, adjusted to β-actin (B,C). Lanes 1–4 are men, lanes 5–8 women, representative of all blots. N = 12 men and 12 women. *P<0.05.

**Figure 5:**
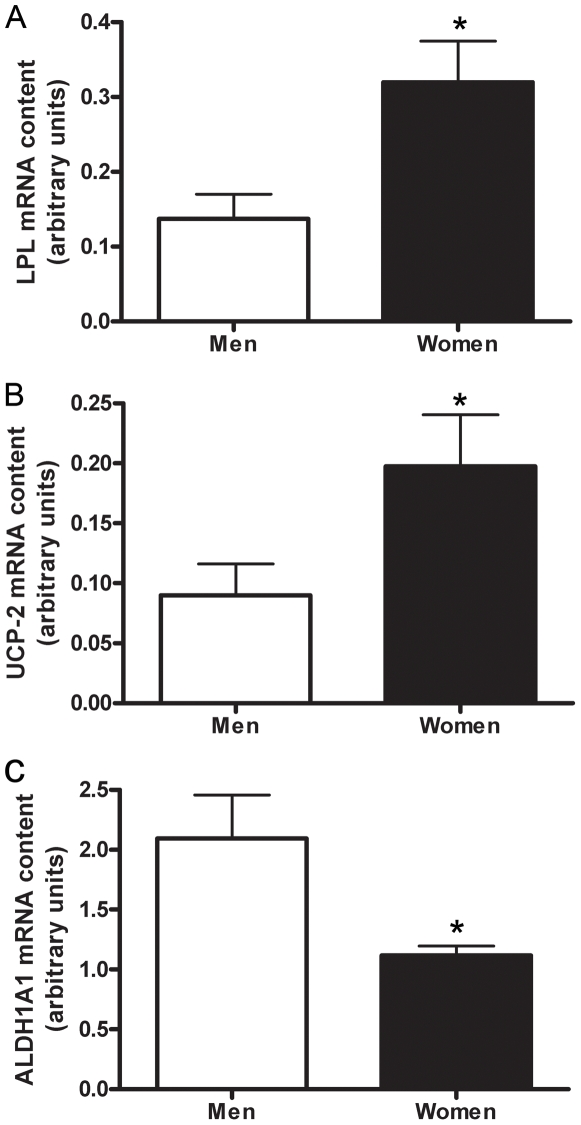
Skeletal muscle mRNA content of LPL, UCP-2 and ALDH1A1 in men and women. LPL is higher in women than men (P = 0.009) (A). UCP-2 is higher in women than men (P = 0.05) (B). ALDH1A1 is lower in women than men (P = 0.01) (C). β2-M mRNA was used as an internal standard. N = 12 men and 12 women.

To determine if sex differences in mRNA content were consistent with protein content we resolved HADHB, ACAA2, and catalase on a western blot and found no significant difference in protein content between men and women (Figure 2c, 3c, 4c;*study 2 samples*).

### Sex difference in muscle fiber composition

Fiber composition data for a subset of *study 1* subjects has been published previously [30]. Fiber composition data for a subset of study 2 subjects was compared to study 1 and when no differences were observed the data was combined to increase the n-value and the data is presented in table 4. Fiber data was consistent with other published data [4], [5], [26], [27] showing women had a significantly higher area% of Type I fibers (women, 32.9±1.3; men, 27.3±1.0; P = 0.001) and a significantly lower area% of Type II fibers (type IIa+type IIx) (women, 67.1±1.3%; men, 72.7±1.0%; P = 0.001) than men (Table 4). Women had significantly smaller mean individual fiber area for type IIa (women, 4777.2±347.8 µm^2^; men, 6066.9±408.3.8 µm^2^; P = 0.013), while there was no significant difference in the type I fiber area or type IIx between women and men (Table 4).

**Table 4:** Fiber type composition in the *vastus lateralis* muscle of women and men.

	Men (N = 10)	Women (N = 16)
**Fiber type composition (area%)**		
Type I	27.3±1.0	32.9±1.3 *
Type IIa+IIx	72.7±1.0	67.1±1.3 *
**Mean area per fiber (µm^2^)**		
Type I	4218.6±225.0	4691.7±477.9
Type IIa	6066.9±408.3	4777.2±347.8 *
Type IIx	5208.7±220.3	4677.7±389.8

Sex differences in fiber type composition between men and women. Due to experimental difficulties data is a combined subset of samples from both study #1 and study#2. Means**±**SEM *P<0.05.

### Sex differences in mRNA content for genes involved in muscle type determination

**Figure 6:**
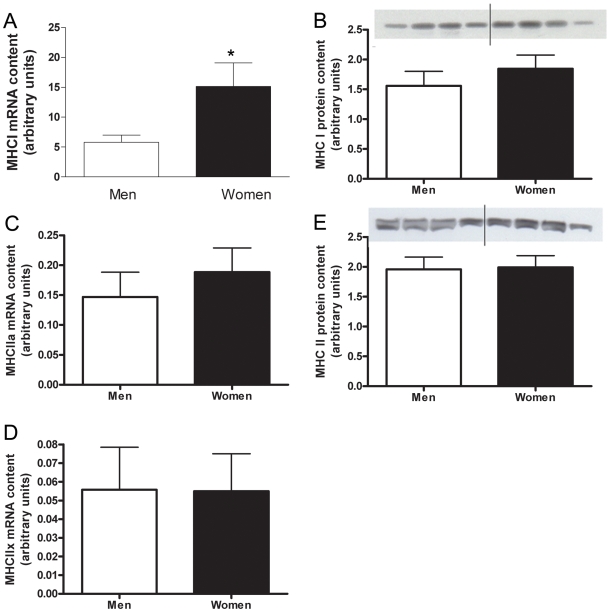
Sex differences in MHC isoforms. Differences in MHC I muscle mRNA content between men and women shown by Real time RT-PCR, adjusted to 28 S rRNA (A). Protein content of MHCI in skeletal muscle of men and women, adjusted to β-actin (B). MHC IIa (C) and MHC IIx (D) muscle mRNA content in men and women shown by Real time RT-PCR, adjusted to 28 S rRNA. Protein content of MHC II in skeletal muscle of men and women (E). Lanes 1–4 are men, lanes 5–8 women, representative of all blots. N = 12 men and 12 women. *P<0.05.

**Figure 7:**
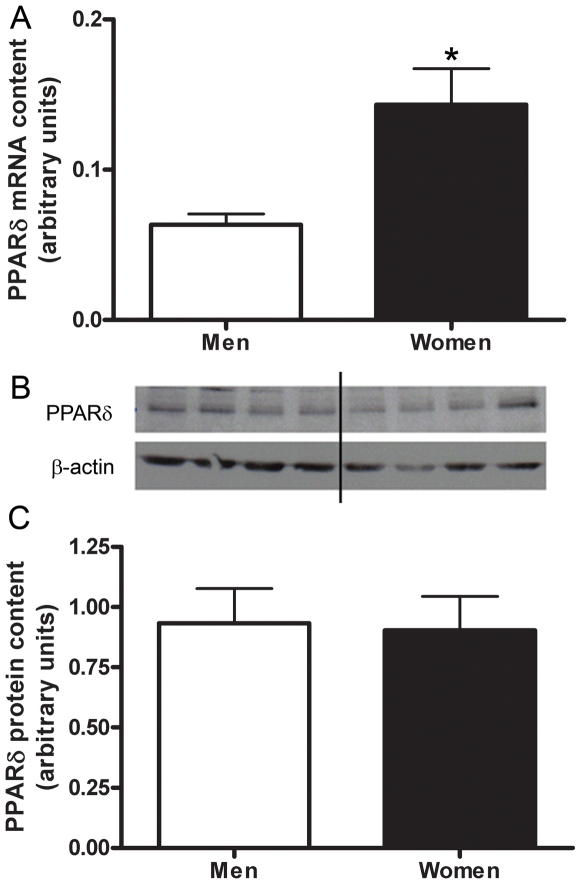
Sex differences in PPARδ. Differences in PPARδ muscle mRNA content between men and women shown by Real time RT-PCR, adjusted to β2-M mRNA (A). Protein content of PPARδ in skeletal muscle of men and women, adjusted to β-actin (B,C). Lanes 1–4 are men, lanes 5–8 women, representative of all blots. N = 12 men and 12 women. *P<0.05.

**Figure 8:**
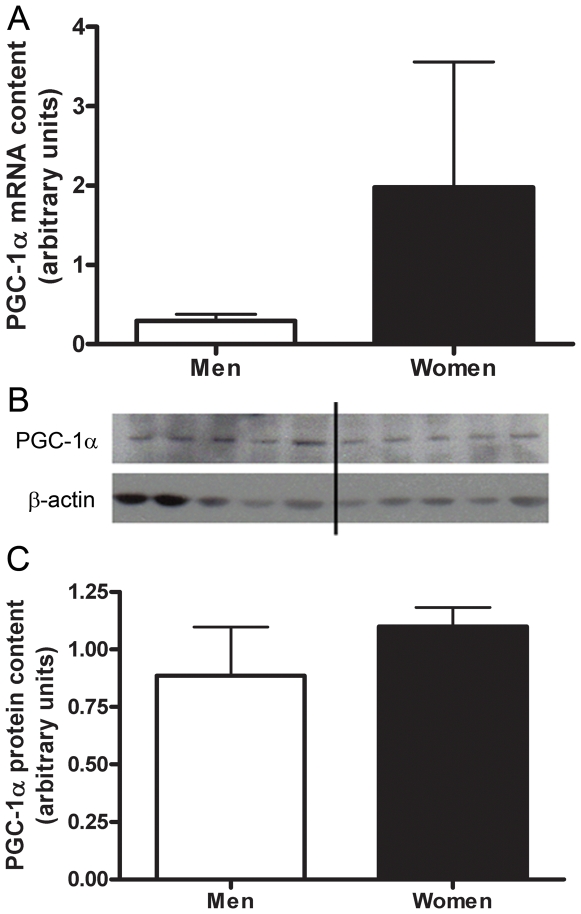
Sex differences in PGC1α. Differences in PGC1α muscle mRNA content between men and women shown by Real time RT-PCR, adjusted to β2-M mRNA (A). Protein content of PGC1α in skeletal muscle of men and women, adjusted to β-actin (B,C). Lanes 1–4 are men, lanes 5–8 women, representative of all blots. N = 12 men and 12 women. *P<0.05.

The stringent microarray analysis revealed no significant genes related to muscle type determination. A more biased targeted RT-PCR approach resulted in the content of MHCI mRNA being significantly higher in the skeletal muscle of women (2.6±0.7 fold, P = 0.035) than men (Figure 6a). No significant difference in the mRNA content of MHCIIa or MHCIIx was found between women and men (Figure 6c,d). Women had a significantly higher mRNA content of PPARδ than men (2.3±0.4 fold, P = 0.004) (Figure 7a). There were no significant differences in the mRNA content of PGC-1α or myostatin in the skeletal muscle of men vs. women (Figure 8a, 9a). Western blot analysis showed no significant sex difference in the protein content of MHC I, MHC II, PPARδ, PGC-1α or myostatin (Figure 6b,e, 7c, 8c, 9c). *All data was acquired from study 2.

**Figure 9:**
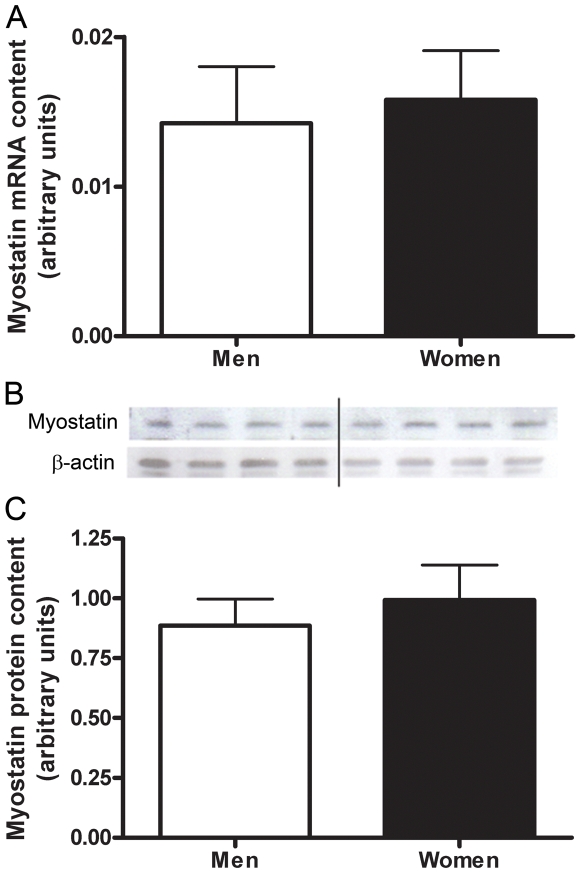
Sex differences in myostatin. Differences in myostatin muscle mRNA content between men and women shown by Real time RT-PCR, adjusted to β2-M mRNA (A). Protein content of myostatin in skeletal muscle of men and women, adjusted to β-actin (B,C). Lanes 1–4 are men, lanes 5–8 women, representative of all blots. N = 12 men and 12 women.

## Discussion

The purpose of this study was to identify novel differences in genes related to metabolism, muscle development, and fiber-type determination and differentiation in mRNA and protein content in skeletal muscle between men and women at rest using microarray, Real Time-PCR analysis, and protein analysis. Microarrays are a useful tool for the identification of novel mRNA expression patterns and can help to understand potential pathways involved in regulating cellular activity in skeletal muscle [33], [38], [45], [46]. The results from these studies indicate that there are significant differences in mRNA content between men and women. Roth et al. (2002) and Welle et al. (2008) have previously shown that there are significant differences in skeletal muscle mRNA content between men and women, as well as showing sex differences are significantly greater than age and/or strength training effects on mRNA content [38], [39]. Approximately 30% of the genes we found to have changed due to sex correspond to the results of Welle and colleagues [39]. Results likely vary between studies due to differences in age of the subject populations, fitness variations, low sample numbers, pooled samples on one gene chip versus individual gene chips per subject, and different gene array technology [38], [39]. The methodology used in this study greatly strengthens the data regarding sex based differences in skeletal muscle mRNA for we examined over 23,000 genes with updated annotation, with 12 subjects per group (N = 24 total) hybridized to individual gene chips for analysis and used stringent statistical analysis with an NLOGP>4. The criteria for differential expression is stringent compared to other array studies that generally use an NLOGP>2, however, necessary for the most accurate unbiased account of gene content differences. For the microarray experiment we used samples from study one which included 7 women in the follicular phase and 5 women in the luteal phase, 6 on oral contraceptives and 6 not on oral contraceptives which gives a good representation of the female population. Given the high n-value of the analysis and stringent array analysis criteria we wanted to reduce any variability in gene expression due to these factors in order to only identify specific and significant gene differences that can be applied to a larger population. The goal of the microarray was not to identify genetic differences due menstrual cycle phase but identify sex related differences between an average population of men and women. In another manuscript “in preparation” from study 2 we used a targeted PCR approach to examine differences in metabolic related genes in men compare to women in both the follicular and luteal phases of the menstrual cycle and found that menstrual cycle had little effect on metabolic related mRNA species, compared to the robust difference that sex has (Figures S1 and S2). Due to these findings, the little amount of precious human muscle sample, and the relatively consistent use of follicular phase women in other gender related studies [5], [11], [17], [18], [19], [20], [47] we compared the mRNA and protein of women in the follicular phase only. It is also important to note that Devries et al (2006) report no physiological difference in exercise performance due to menstrual cycle phase including average RER, glycogen utilization, glucose rate of appearance, rate of disappearance, and metabolic clearance rate averaged over the exercise period [41].

The first focus was on the mRNA content of genes involved in intermediary substrate metabolism due to the known fact that women oxidize more fat during endurance exercise as compared with men [5], [6], [7], [8], [10], [11], [48]. Array results identified six genes related to metabolism that were differentially regulated between men and women. We chose to confirm the mRNA content of five of these genes by RT-PCR. Results identified a novel sex-based difference in the mRNA content of ACAA2 and HADHB (TFP-β). Also, the mRNA content for catalase, lipoprotein lipase, and uncoupling protein-2 were higher in women compared with men. Lastly, ALDH1A1 mRNA content was lower in women compared with men. There was also sex based differences in the mRNA content of genes involved in protein biosynthesis, cell proliferation, signal transduction, transcription, and translation with a particular interest in those genes which are directly involved in muscle function and/or structure.

HADHB (TFPβ) is a multi-enzyme complex found in the mitochondria that is involved in the β-oxidation of fatty acids [49]. Specifically, the TFP enzyme catalyzes the last three steps of long chain fatty acid β-oxidation for long-chain specific acyl-CoA moieties. We are the first to measure and report a sex difference in the mRNA content for this gene. An enhanced β-oxidation capacity distal to transport of FFA into the mitochondria may allow for maintenance of β-oxidation when the cell is under metabolic stress. For example, male transgenic peripheral peroxisome activating receptor knockout (PPARα^−/−^) animal develop severe hypoglycemia when an inhibitor of CPT activity (etomoxir) was given, yet the majority of female mice survived [50]. Furthermore, CPTII deficiency is an autosomal recessive condition and yet many more cases have been documented in men as compared with women, possible due to enhanced β-oxidation capacity in women. Others have reported that the short-chain specific isoform of HAD (SCHAD) has higher mRNA and protein abundance in women compared with men [2], [51].

These results also indicate a significant sex based difference in the mRNA content of ACAA2 (acetyl-Coenzyme A acyltransferase 2). ACAA2 is one of two isoforms of ACAA, which is an intracellular enzyme that biosynthesizes cholesteryl esters. Specifically, ACAA is involved in storing cholesteryl esters as lipid droplets, in absorbing dietary cholesterol, and in providing cholesteryl esters as part of the core lipid for lipoprotein synthesis and assembly [52], [53], [54]. Although ACAA appears to be present in many cell types such as hepatocytes, adrenal cells, skin cells, intestinal enterocytes, neurons, and macrophages it has not been well characterized in skeletal muscle [55], [56]. In the aforementioned cell types it has been shown that the ACAA1 isoform is the predominant enzyme compared to ACAA2 [55]. Our gene array results suggest that men and women have no significant difference in the expression of ACAA1 but women have an increased expression of ACAA2 compared to men. Therefore, the total ACAA expression should be significantly higher in women than men, which may explain why lipids are more readily available for substrate utilization during endurance exercise in women. Future studies are needed to determine the expression profile of ACAA1 and ACAA2 in human skeletal muscle as well as the importance of ACAA2 over-expression in women compared to men.

Catalase is one of the three primary antioxidant enzymes. RT-PCR confirmed that mRNA content for catalase is significantly higher in women compared to men. Interestingly, Fano et al (2001) have shown that the enzyme activity of catalase, in the *vastus lateralis*, is significantly higher in women than men [57]. Sex differences have also been observed in other antioxidant enzyme, including manganese-superoxide dismutase [58], suggesting that women are better protected against reactive oxygen species (ROS) as compared with men. We also demonstrated that mRNA content for UCP2 was higher in women compared to men. UCP's can affect energy metabolism efficiency by uncoupling ATP production from mitochondrial respiration. UCP2 is involved in the regulation of energy metabolism and might play a role in obesity [59], [60], [61]. UCP2 has also been suggested to affect the production of reactive oxygen species (ROS) [62], [63], [64], and regulate the [ATP]/[ADP] ratio [64], [65], [66]. Although we are the first to show a sex specific difference in mRNA content in the *vastus lateralis*, this may help to explains why young women are protected against ROS (reviewed in [67]). Furthermore, if UCP2 does play a role in obesity the higher expression in women might help to regulate lipid oxidation.

ALDH1A1 is an isoform of the aldehyde dehydrogenase superfamily primarily responsible for the oxidation of endogenous and exogenous aliphatic and aromatic aldehydes including acetaldehyde, benzaldehyde, 4-hydroxynonenal, malondialdehyde, and retinaldehyde [68], [69], [70]. Recently ALDH1A1 was also shown to convert 3-deoxyglucosone into 2-keto-3-deoxygluconate [71]. In this study we found that the mRNA content was lower in women compared with men, suggesting that men might be able to metabolize aldehydes (i.e. alcohol) more efficiently than women. ALDH1A1 has not been well characterized in humans, let alone skeletal muscle, but in the mouse liver and human colon there appears to be no sex differences in activity [72], [73]. Further studies into the implications of ALDH1A1 mRNA sex differences in skeletal muscle need to be conducted.

These results also showed sex specific differences in genes involved in cell transport, protein biosynthesis, cell proliferation, signal transduction pathways, transcription and translation. Of interest, women had a 2.2-fold reduction in the solute carrier family 1, member 4 that is involved in glutamate/neutral amino acid transport which could be important in metabolism. Women had a 1.7-fold increase in mRNA content of angiopoietin 1, a factor involved in increasing vascularization to a specific tissue, in this case, muscle; however, there does not seem to be a significant difference in capillarization of the *tibialis anterior* or *vastus lateralis* muscle of women compared to men [74], [75]. Women also had a 1.7- and 1.5-fold reduction in Dishevelled associated activator of morphogenesis 2, and spectrin- beta- non-erythrocytic 1 which are involved in actin cytoskeleton organization and biogenesis, and barbed-end actin filament capping, respectively. Both are important in muscle cell shape and function. There was an interesting trend in the expression of signal transduction genes and transcription factor genes. Women had a significant up-regulation of the majority of signal transduction related genes and a significant down-regulation of the majority of transcription factor genes. Despite these trends, there was no evidence that one signal transduction pathway was favored over another as the identified genes spanned multiple pathways including the MAPK pathway, frizzled signaling pathway, G-protein coupled receptor protein signaling pathway, and insulin receptor signaling pathway.

It is important to note that gene array analysis works on the assumption that basal mRNA equates into changes at the protein level and thus activity level. However, recent studies have been demonstrating that is not always the case. For example Kiens et al. (2004) demonstrated that women have a significantly higher LPL mRNA content; however there was no observed differences in LPL activity between men and women [47]. Similarly, Roepstorff et al. demonstrated that although mRNA and protein expression of hormone sensitive lipase (HSL) was higher in women, phosphorylation activation was significantly higher in men [11]. Another study in skeletal muscle biology also found discrepancies in the correlation between mRNA and protein content of a number of genes related to fatty acid oxidation [76]. Part of the discrepancy between mRNA abundance and protein and enzyme assays may relate to higher variance in Western blots and activity assaystechnique, and/or that the transcriptome abundance regulates multiple interacting and synergistic pathways that combine to influence flux through metabolic pathways at the protein level that is below the detectable threshold for statistical changes in a single given protein to be manifested. In order to fully understand cellular differences between men and women it is important to understand pre-translational (mRNA abundance), translational (protein) and post-translational (phosphorylation) levels of control.

It has recently been hypothesized that some of the sex differences in exercise substrate selection may be due to fiber-type compositional differences [32]. Subject fiber type characteristics were the same in this study as previously reported [4], [5], [26], [27], [30], [32]; specifically, the proportionate area (area%) of type I fibers was higher, while that of type II fibers was lower, in women compared with men. Previous findings of a sex difference in type I fiber proportion [25], [28], [29], [32] and a larger type I fiber area [4], [5], [27], [30], in women as compared with men were not confirmed. Nevertheless, it is the proportion of the total muscle area represented by a given fiber type (area X proportion = area%) that should determine the overall abundance of a given transcript or protein in a homogenate of skeletal muscle. Examination of mRNA expression of myosin heavy chain genes, which are specifically expressed in their corresponding muscle fiber types [77], are good markers of the terminal differentiation of muscle fibers. In this study, we found a significantly higher mRNA content of MHCI and a similar mRNA content of MHCIIa and MHCIIx in the skeletal muscles of women compared with men. The difference in MHCI mRNA did not translate into differences in MHCI protein expression, consistent with previous findings [30].

Similarly, we found sex differences in the mRNA content, but not the protein content, of PPARδ; which plays a role in the conversion of muscle fiber type II into type I and maintenance of the number of type I fibers [78], and increases the capacity for oxidative metabolism of muscle fibers through hyperplasia of type I fibers [79], [80] in transgenic mice.

Strong evidence in transgenic mice [81], and controversial evidence in humans [82], [83], [84], suggests that PGC-1α is important in the determination of muscle fiber type and induces a fiber type transformation from type II into type I muscle fibers. We did not find an influence of sex on the mRNA or protein content of PGC-1α in skeletal muscle in spite of the fact that women had a higher% area of type I fibers. We also found that there was no sex difference in the mRNA content of myostatin, a negative regulator of skeletal muscle growth [85], [86], [87].

At rest, there are no significant differences in protein content of the select genes examined, which are involved in metabolism or fiber type. Consistently, there are no observed sex differences in substrate utilization at rest [7], [8], [88], [89], [90]. However, mRNA content suggest that men and women are “primed” differently for specific cellular events, and future studies are need to determine if exercise induces changes at the translational and post-translational levels.

Overall, these results identified sex-based differences in mRNA content of metabolic related genes that might lead the way towards an understanding of the sex-based differences in metabolic fuel selection during endurance exercise. Furthermore, this study emphasizes the importance of the influence of sex based differences in gene expression. At the mRNA level there are no inconsistencies in our data or in the literature, which supports that women have higher mRNA abundance for genes involved in fat metabolism as compared with men. Furthermore, men and women demonstrate varied regulation of genes involved in mitochondrial function, transport, protein biosynthesis, cell proliferation, signal transduction pathways, transcription and translation, even at rest.

## Supporting Information

Table S1 Affymetrix gene array analysis comparing resting human skeletal muscle of women with men. Original Affymetrix data. LogFC; Log fold-change, NLogP;negative log of the p value (>4.0), F; Woman M; Man.
(0.04 MB XLS)

Figure S1 Menstrual cycle differences in resting mRNA content of genes related to substrate metabolism. Genes are expressed as mean fold difference follicular/luteal±SEM. β2-M mRNA was used as an internal standard. N = 12 men and 12 women. *P<0.05.
(1.37 MB TIF)

Figure S2 Sex differences in resting mRNA content of genes related to substrate metabolism. Genes are expressed as mean fold difference women/men±SEM. β2-M mRNA was used as an internal standard. N = 12 men and 12 women. *P<0.05.
(1.83 MB TIF)
