# Supplementary figures and images for: Sex Differences in Global mRNA Content of Human Skeletal Muscle
Source: PLoS One. 2009 Jul 22;4(7):e6335. doi: 10.1371/journal.pone.0006335 (PMC2709437; doi:10.1371/journal.pone.0006335)

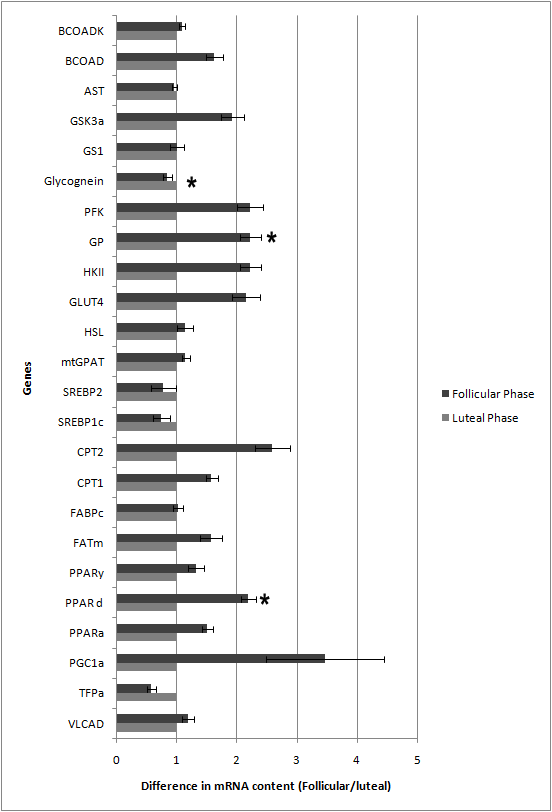

Supplement: Figure S1 — Menstrual cycle differences in resting mRNA content of genes related to substrate metabolism. Genes are expressed as mean fold difference follicular/luteal±SEM. β2-M mRNA was used as an internal standard. N = 12 men and 12 women. *P<0.05. (1.37 MB TIF) [file pone.0006335.s002.tif]

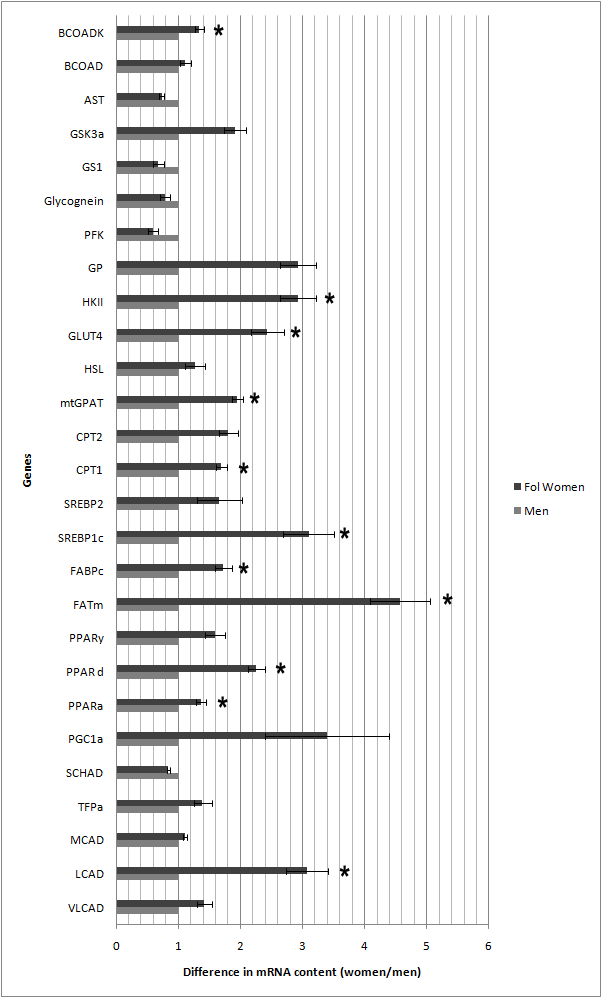

Supplement: Figure S2 — Sex differences in resting mRNA content of genes related to substrate metabolism. Genes are expressed as mean fold difference women/men±SEM. β2-M mRNA was used as an internal standard. N = 12 men and 12 women. *P<0.05. (1.83 MB TIF) [file pone.0006335.s003.tif]
